# Targeting CBP revers chemoresistance to 5‐FU of CDX2/REG4 double‐positive gastric cancer

**DOI:** 10.1002/ctm2.70069

**Published:** 2024-10-25

**Authors:** Zhiyuan Fan, Fangyuan Li, Xiao Jiang, Tao Pan, Mingde Zang, Jianfang Li, Beiqin Yu, Qingqing Sang, Wentao Liu, Liping Su, Chen Li, Zhenggang Zhu, Min Yan, Chao Yan, Fei Yuan, Bingya Liu

**Affiliations:** ^1^ Shanghai Key Laboratory of Gastric Neoplasms, Shanghai Institute of Digestive Surgery, Ruijin Hospital, Shanghai Jiao Tong University School of Medicine Shanghai China; ^2^ Department of Breast Surgery Shanghai First Maternity and Infant Hospital, School of Medicine, Tongji University Shanghai China; ^3^ Department of General Surgery Ruijin Hospital, Shanghai Jiao Tong University School of Medicine Shanghai China; ^4^ Department of Pathology Shanghai Tenth People's Hospital, Tongji University School of Medicine Shanghai China; ^5^ Department of Gastric Cancer Surgery, Department of Oncology Fudan University Shanghai Cancer Center, Shanghai Medical College, Fudan University Shanghai China; ^6^ Department of Pathology Ruijin Hospital, Shanghai Jiao Tong University School of Medicine Shanghai People's Republic of China

Dear Editor,

We conducted a study exploring the potential of cyclic‐AMP response element binding protein (CBP) inhibitors in overcoming the chemoresistance of CDX2/REG4 double‐positive gastric cancer (GC) to 5‐FU chemotherapy.

CDX2 is a classical transcription factor belonging to the caudal‐related homeobox gene family, which determines the development and maintenance of intestinal differentiation in the gut and is overexpressed  in part of GC.^1^ Our study aims to investigate the heterogeneity of CDX2+ GC, which accounts for approximately 50% of all GC,^1^ and discover potential therapies. Using 111 GC samples, molecular classification based on CDX2 expression revealed that CDX2+ GC could be further divided into two subtypes: REG4^hi^ and REG4^lo^ (Figure 1A). REG4 is a direct target of CDX2 and has been implicated in the progression and chemoresistance of GC.^2^ The REG4^hi^ subtype showed significantly shorter overall survival (OS, Figure 1B, hazard ratio: CDX2^hi^ REG4^hi^ vs. CDX2^lo^ .99, 95% CI .60–1.64, *p* = .973; CDX2^hi^ REG4^lo^ vs. CDX2^lo^ .11, 95% CI .04–.30, *p* < .001; CDX2^hi^ REG4^lo^ vs. CDX2^hi^ REG4^hi^ .12, 95% CI .04–.30, *p* < .001) and poorer differentiation compared to REG4^lo^ (Figure 1C, Tables S1 and S2). REG4 positive expression was significantly associated with CDX2+ cases (Figure 1D). Additionally, CDX2+ REG4^hi^ GC patients were more resistant to 5‐FU‐based chemotherapy (Figure 1E). We identified CDX2+ GC cell lines (Figure 1F) with high or low REG4 expression (Figure 1G) using the CCLE database. The IC_50_ of CDX2+ REG4^hi^ GC cells to 5‐FU were much higher than those of CDX2+ REG4^lo^ GC cells (Figure 1H–J).

**FIGURE 1:**
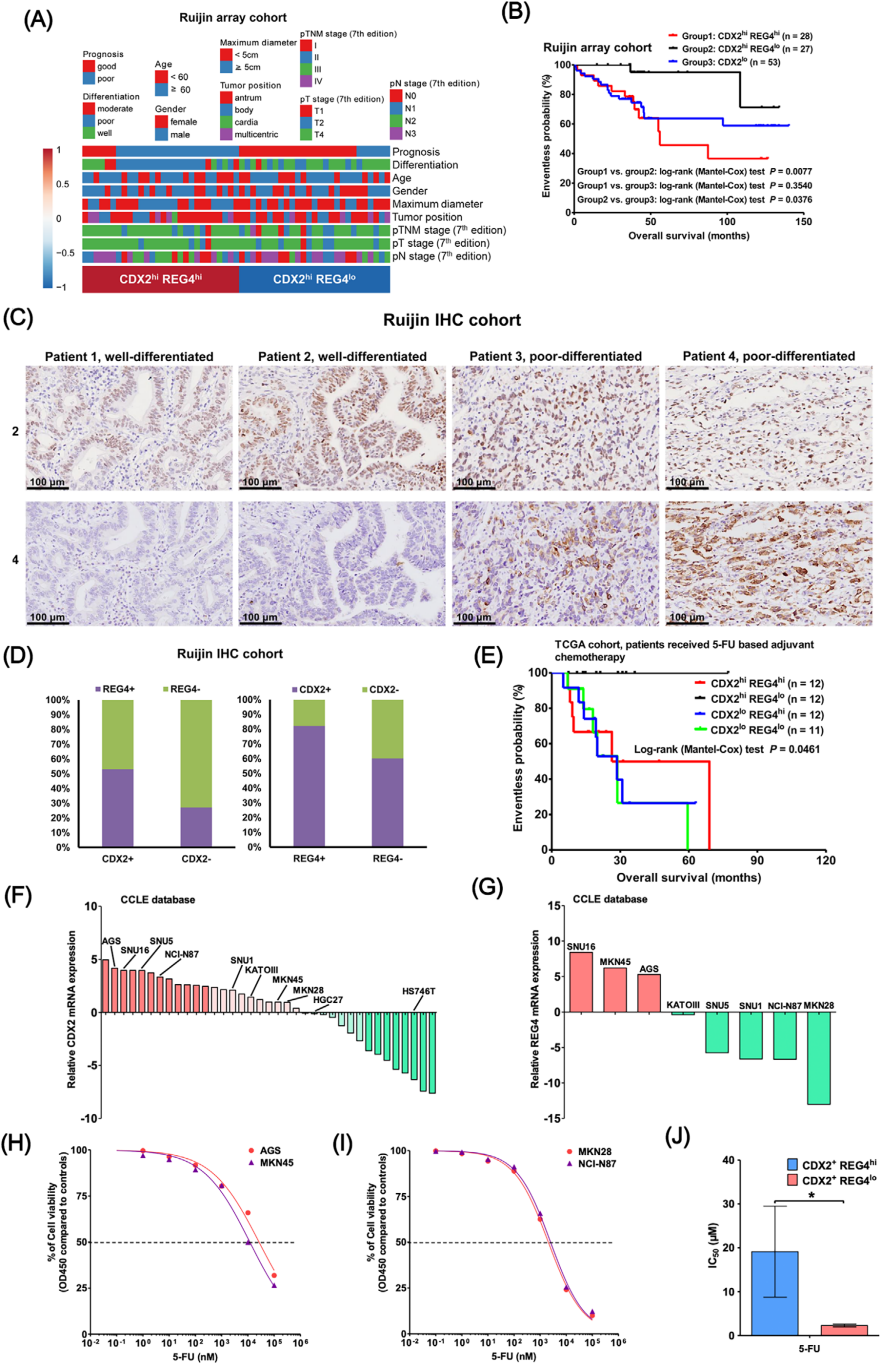
REG4 is a powerful prognostic stratification factor for CDX2^+^ GC. (A) 111 GCs from the Ruijin array cohort were classified into CDX2^hi^ (*n* = 56) and CDX2^lo^ (*n* = 55) groups according to median expression level of CDX2. Association of patient clinicopathological features with the two intrinsic subtypes of CDX2^hi^ GC by heatmap. Patient prognosis and tumour differentiation were the only two clinicopathological parameters significantly related to the two subtypes (*p* < .01 for both parameters; *χ* ^2^ test). (B) Kaplan–Meier plots for the OS of the patients with CDX2^hi^ REG4^hi^ (*n* = 28), CDX2^hi^ REG4^lo^ (*n* = 27) and CDX2^lo^ GC (*n* = 53). One sample from a patient with CDX2^hi^ GC and two samples from patients with CDX2^lo^ GC without follow‐up information were not included in the survival analysis. (C) CDX2 and REG4 protein expression in 161 GC samples (the Ruijin IHC cohort) were detected by immunochemistry (IHC) staining. Representative images of CDX2 (the upper) and REG4 (the lower) expression patterns. (Original magnification, ×200 for all images.) (D) Association between the expression of CDX2 and REG4. Left, proportions of REG4^+^ and REG4^−^ cases in CDX2^+^ and CDX2^−^ GC. Right, proportions of CDX2^+^ and CDX2^−^ cases in REG4^+^ and REG4^−^ GC. For all Kaplan–Meier plots, *p* values were obtained using the log‐rank (Mantel‐Cox) test and the + symbols indicate censored data. (E) Kaplan–Meier plots for the OS of 47 patients who received 5‐FU‐based adjuvant chemotherapy. The 47 GC patients were further classified into the following four molecular subtypes: CDX2^+^ REG4^+^ (*n* = 12), CDX2^+^ REG4^−^ (*n* = 12), CDX2^−^ REG4^+^ (*n* = 12) and CDX2^−^ REG4^−^ (*n* = 11). Kaplan–Meier plots for the OS of the four subtypes were compared. For all Kaplan–Meier plots, *p* values were obtained using the log‐rank (Mantel‐Cox) test and the + symbols indicate censored data. (F) CDX2 mRNA levels in the commonly used GC cell lines in the CCLE database (https://portals.broadinstitute.org/ccle). Red and green colours represent high and low expression levels of CDX2, respectively. (G) REG4 mRNA levels in the 8 CDX2^+^ GC cell lines conserved in our laboratory. Data are presented as the means ± SD. Error bars represents for standard deviations. (H‐I) Dose‐response curves for the CDX2+ REG4hi (H) and CDX2+ REG4lo (I) subtype GC cell lines treated with the indicated doses of 5‐FU for 72 hours. (J) Comparison of the IC50s of the two subtypes of CDX2+ GC cell lines treated with ‐5FU. *p < .05.

We selected GC cell lines which showed consistent expression patterns of CDX2 and REG4 with specific GC types suggested by CCLE database and confirmed by immunoblotting (Figure 2A). A screen of 17 small molecule inhibitors targeting epigenetic regulators (Table S3) identified CPI‐637, a CBP/p300 inhibitor, as particularly effective against CDX2+ REG4^hi^ GC cells (Figure 2A and B). These cells showed significant growth inhibition (Figure 2C) and lower IC50 values (Figure 2D) with CPI‐637 compared to CDX2+ REG4^lo^ cells. In vivo experiments demonstrated that CPI‐637 significantly inhibited tumour growth in CDX2+ REG4^hi^ cell derived xenograft CDX (Figure 2E), resulting in smaller tumour volumes (Figure 2F), less tumour weight (Figure 2G) and higher tumour growth inhibition rates (Figure 2H).

**FIGURE 2:**
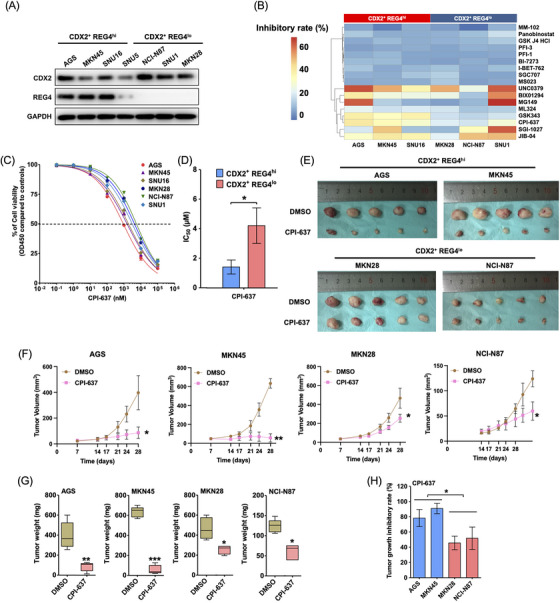
CDX2^+^ REG4^hi^ GC cells are more sensitive to CPI‐637. (A) Western blot of CDX2, REG4 and the loading control GAPDH in 7 CDX2^+^ GC cell lines. (B) Identification of CPI‐637 as an inhibitor selectively against CDX2^+^ REG4^hi^ GC cells. CDX2^+^ REG4^hi^ GC cells (AGS, MKN45 and SNU16) and CDX2^+^ REG4^lo^ GC cells (MKN28, NCI‐N87 and SNU1) were treated with the indicated epigenetic small molecule inhibitor with an IC_50_ concentration of its target activity or an equal volume of. 1% DMSO for 72 h and then assayed for growth with CCK‐8 proliferation assays. The heatmap shows the growth inhibition rates of the 17 inhibitors on each cell line compared with the same cell line treated with DMSO. Red and blue colours represent relatively high and low growth inhibition rates, respectively, as indicated in the scale bar. (C) Dose‐response curves for the two subtypes of CDX2^+^ GC cell lines treated with the indicated doses of CPI‐637 for 72 h in in vitro culture. (D) The IC_50_s of CPI‐637 in the two subtypes of CDX2^+^ GC cell lines, two‐tailed unpaired Student's *t* test. (E–G) Suppression of in vivo growth of the two subtypes of CDX2^+^ GC cell lines by CPI‐637. Mice carrying AGS, MKN45, MKN28 or NCI‐N87 xenografts were treated with CPI‐637 (25 mg/kg per dose) or equal volume of vehicle (DMSO) intraperitoneally three doses per week (*n* = 5 mice per group). (E) Images showing representative tumour volumes of each treatment group of xenograft models. (F) Tumour growth curves of the indicated treatment groups. Tumour size was assessed twice a week via vernier callipers, and tumour volume was calculated, two‐way ANOVA test. (G) Comparing tumour weights of the indicated treatment groups of each xenograft model at the time of sacrifice, two‐tailed unpaired Student's *t* test. (H) Comparison of in vivo tumour growth inhibition rates of the two subtypes of CDX2^+^ GC cell lines by CPI‐637, two‐tailed unpaired Student's *t* test. Data are presented as the means ± SD. Error bars represents for standard deviations. *p < .05, **p < .01, ***p < .0001, *NS*, not significant.

CPI‐637 is a selective inhibitor targeting both CBP and p300^3^ and its role have been investigated in tumour treatment.^4^, ^5^ CBP/p300 activates gene expression using its protein lysine acetyltransferase (KAT) domain to catalyse the histone H3 lysine 18 or lysine 27 acetylation (H3K18Ac or H3K27Ac) and its bromodomain to recognise acetyl‐lysine residues in histone tails.^6^ AGS and MKN28 had similarly high levels of CBP and silenced expression of p300 (Figure S1A) probably due to EP300 nonsense mutations (Figure S1B). p300 showed relatively low expression in MKN45 and NCI‐N87 cells with no mutations (Figure S1A). We further found that H3K18Ac/H3K27Ac decreased upon CPI‐637 treatment in AGS and NCI‐N87 cells was in a dose‐dependent manner (Figure S1C and D). Significant reductions in H3K18Ac/H3K27Ac levels with 5 µM CPI‐637 were observed in GC cell lines (Figure S1C–E). CPI‐637 impaired REG4 expression in CDX2+ REG4^hi^ GC cells but did not affect REG4 expression in CDX2+ REG4^lo^ ones (Figure S1E and F).

REG4 was identified as a direct target of CDX2 in a previous study.^2^, ^7^ Two previously reported CDX2‐binding sites on the REG4 promoter located near the transcriptional start site (TSS) (Figure 3A). ChIP analyses revealed that H3K27Ac was abundant on the REG4 promoter in control‐treated CDX2+ REG4^hi^ AGS cells (Figure 3B), while CPI‐637 treatment induced a transition to H3K27Me3 (Figure S2A). Conversely, in CDX2^+^ REG4^lo^ NCI‐N87 cells, H3K27Me3, rather than H3K27Ac or H3K18Ac, was specifically enriched on the *REG4* promoter, which was not affected by CPI‐637 treatment (Figure S2B). Co‐IP experiments showed that CDX2 physically interacted with CBP (Figure 3C), and this interaction was disrupted by CPI‐637 treatment or CDX2 silencing (Figure 3D and E), indicating that the regulation of REG4 by CBP was CDX2‐dependent.

**FIGURE 3:**
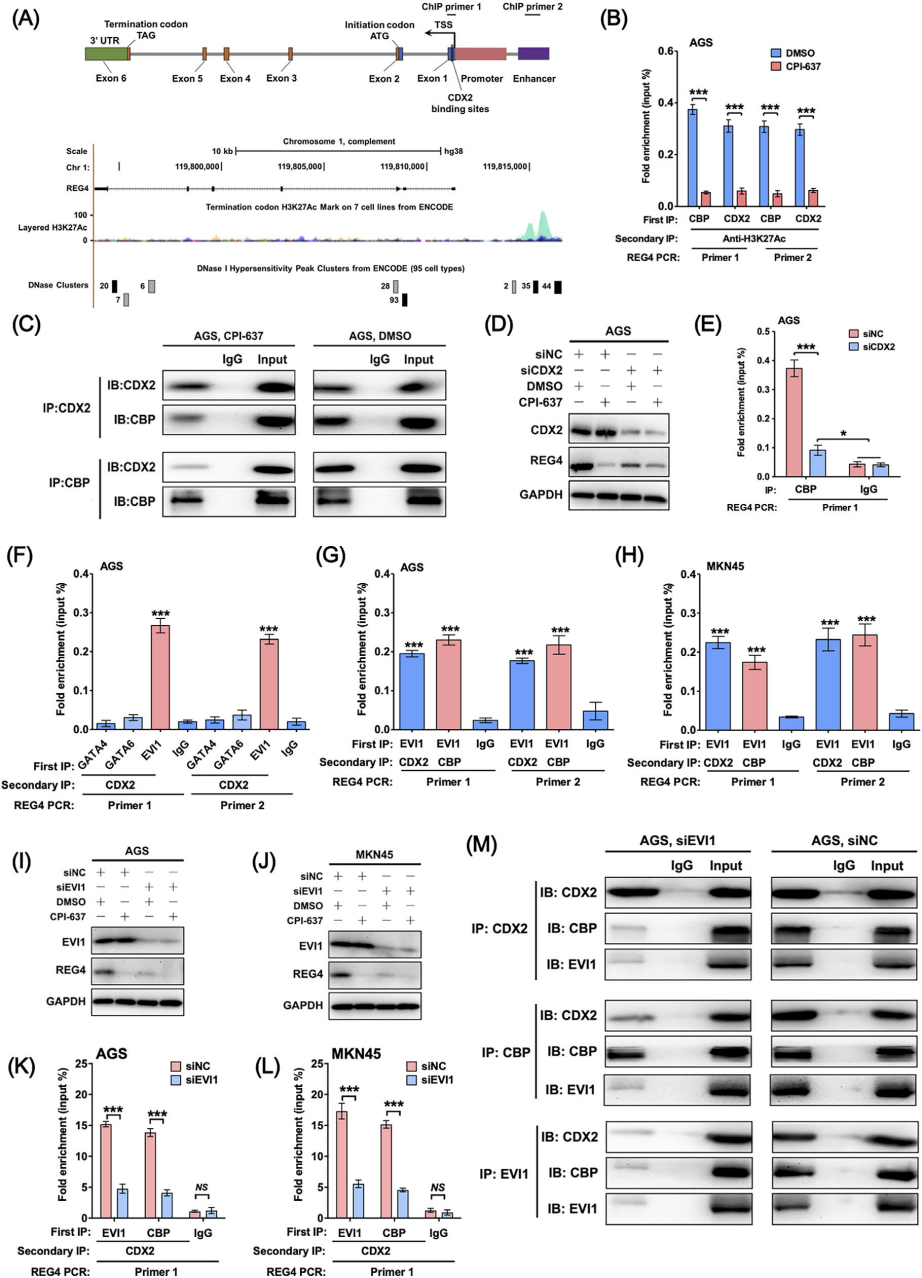
Selective recruitment of CBP to the *REG4* promoter by CDX2 in CDX2^+^ REG4^hi^ GC cells requires EVI1. (A) Analysis of the genomic context of *REG4* using the UCSC Genome Browser (http://genome.ucsc.edu/). The two previously reported CDX2‐binding sites (Chr1:‐119811549 ∼ ‐119811543 (hg38/Human), 5′‐AATAATA‐3′; and Chr1:‐119811574 ∼ ‐119811567 (hg38/Human), 5′‐TTTTATGG‐3′) on the *REG4* promoter are located near the transcriptional start site (TSS). The ENCODE ChIP‐seq dataset annotates a H3K27Ac peak region with DNase clusters (candidate enhancer region) upstream of the *REG4* gene body (approximately 4 kb from the TSS). ChIP primers 1 and 2 represent the sites to that amplify the CDX2‐binding motif regions (promoter) and the enhancer region in ChIP experiments, respectively. (B) Sequential ChIP in AGS cells. Cells were treated for 24 h, and then protein‐DNA was cross‐linked and sequentially immunoprecipitated by two antibodies as indicated. The purified DNA fragments were amplified and quantified by qPCR using the promoter and enhancer primer pairs described in (A). Enrichment of the indicated protein on the *REG4* transcriptional regulatory regions was shown as a percentage of the input (% input). (C) Co‐immunoprecipitation (Co‐IP)‐Western blot for detecting the physical interactions between endogenous CDX2 and CBP with or without 5 µM CPI‐637 treatment in AGS cells for 24 h. IgG, immunoglobulin G. IB, immunoblot. (D) AGS cells were transiently transfected with a siRNA cocktail targeting CDX2 (siCDX2) or the negative control siRNA (siNC) for the first 24 h, followed by treatment with or without 5 µM CPI‐637 in AGS cells for another 48 h. The expression levels of CDX2, REG4 and the loading control GAPDH in AGS cells were detected by Western blot. (E) Enrichments of CBP on the *REG4* promoter in AGS cells were detected by ChIP. (F–H) Sequential ChIP in AGS (F and G) and MKN45 (H) cells. Protein‐DNA was cross‐linked and sequentially immunoprecipitated by two antibodies as indicated. IgG was used as a negative control. The purified DNA fragments were amplified and quantified by qPCR using the promoter and enhancer primer pairs, respectively. Enrichment of the indicated protein on the *REG4* transcriptional regulatory regions was shown as a percentage of the input (% input). (I–L) AGS and MKN45 cells were transiently transfected with siRNA targeting EVI1 (siEVI1) or the negative control siRNA (siNC) for 24 h and then treated with or without 5 µM CPI‐637 for another 48 h. Then, the expression levels of EVI1, REG4 and the loading control GAPDH in AGS (I) and MKN45 (J) cells was detected by Western blot. Enrichment of CDX2, EVI1 and CBP on the promoter of *REG4* in AGS (K) and MKN45 (L) cells were detected by sequential ChIP, two‐tailed unpaired Student's *t* test. (M) Co‐immunoprecipitation (Co‐IP)‐Western blot for detecting the physical interactions between the indicated proteins in AGS cells transiently transfected with siEVI1 or siNC for 72 h. IgG, immunoglobulin G. IB, immunoblot. Data are presented as the means ± SD. Error bars represents for standard deviations. *p < .05, **p < .01, ***p < .0001, *NS*, not significant.

GSEA in our GSE54129 cohort revealed GATA4, GATA6 and ecotropic viral integration site‐1 (EVI1) were most significantly enriched in CDX2^+^ REG4^hi^ GC (Figure S2C, all FDR *q* value < .001). A total of 168 differentially expressed genes (DEGs) between two subtypes of CDX2^+^ GC were identified among GSE54129 and two other independent GC cell line cohorts (GSE15455 and GSE22183, Figure S2D, Table S4). EVI1 was found to co‐occupy the REG4 promoter with CDX2 and CBP (Figure 3F and G). EVI1 silencing abrogated REG4 expression (Figure 3H and I) and blocked the interaction between CDX2 and CBP (Figure 3J–L), indicating that EVI1 was crucial for the recruitment of CBP to the REG4 promoter by CDX2. GATA4 and GATA6 were not significantly differentially expressed between the two GC cell subtypes (Figure S2E). Sequential ChIP analyses showed none of the three transcriptional factors (TFs) occupied the REG4 promoter in MKN28 and NCI‐N87 cells (Figure S2F).

CBP can catalyse lysine acetylation via its KAT activity.^8^, ^9^ We found that the EVI1 acetylation level was high in CDX2+ REG4^hi^ AGS cells (Figure S2G) and CBP silencing by siRNA nearly abrogated EVI1 acetylation (Figure S2G). The region between amino acids 283 and 514 of the EVI1 protein was reported to contribute to the interaction between EVI1 and CBP.^10^ We identified three candidate CBP KAT‐specific lysine acetylation sites (lysine 359, 421 and 425) in this EVI1 region using the GPS‐PAIL algorithm^9^ (Figure S2H and I). AGS cells with silenced endogenous EVI1 were reintroduced with the wild‐type EVI1 (EVI1 WT) or three EVI1 mutants (EVI1 K359R, EVI1 K421R, or EVI1 K425R) (Figure S2J). Co‐IP analyses showed that the mutation of K421 rather than K359 and K425 significantly attenuated CBP‐induced EVI1 acetylation (Figure S2K). The mutation of EVI1 K421 to arginine abrogated the physical binding of EVI1 to CBP or CDX2 and promoted the recruitment of the corepressor C‐terminal binding protein 1 (CtBP1) to EVI1 (Figure S2L). S2

We also analysed whether the application of the CBP inhibitor could overcome the 5‐FU resistance of CDX2^+^ REG4^hi^ GC cells. CPI‐637 and 5‐FU combination treatment significantly improved the efficacy of 5‐FU or CPI‐637 alone in AGS and MKN45 cells (Figure 4A and B). The calculation of combination index (CI) values showed that CPI‐637 and 5‐FU combination had a synergistic effect in CDX2^+^ REG4^hi^ AGS and MKN45 cells (Figure 4C), but the addition of CPI‐637 didn't improve the effect of 5‐FU in CDX2^+^ REG4^lo^ MKN28 and NCI‐N87 cells (Figure 4D).

**FIGURE 4:**
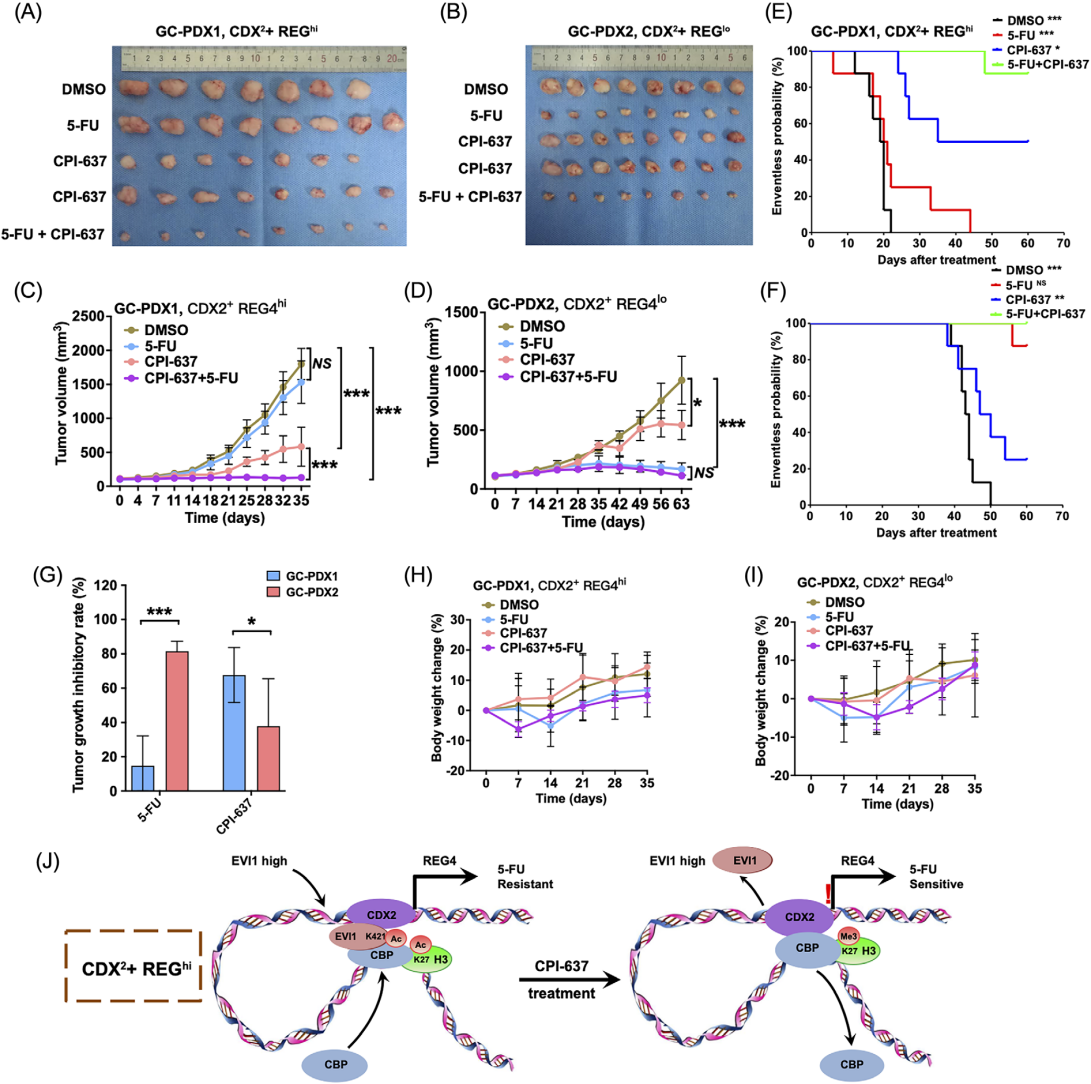
CPI‐637 Increases CDX2^+^ REG4^hi^ GC Sensitivity to 5‐FU. (A–I) GC‐PDX1 (CDX2^+^ REG4^hi^) and GC‐PDX2 (CDX2^+^ REG4^lo^) mice (A and B) were treated with DMSO, 5‐FU (20 mg/kg per dose) and CPI‐637 (25 mg/kg per dose) alone and a combination of 5‐FU and CPI‐637 intraperitoneally at three doses per week for the indicated number of days (*n* = 16 for CPI‐637 group and *n* = 8 mice per group for other groups). (A and B) Images showing representative tumour volumes of each treatment group of PDX models. (C and D) Tumour growth curves of the indicated treatment groups, two‐way ANOVA test. (E and F) Eventless probability curves of the indicated treatment groups. (G) Comparison of tumour growth inhibitory rates of GC‐PDX1 and GC‐PDX2 in the indicated treatment groups, two‐tailed unpaired Student's *t* test. (H and I) Body weight change curves of the indicated treatment groups. Body weight was measured every two weeks by the balance and the percentage of weight change was calculated. (J) Schematic diagram showing the mechanisms of forming the two intrinsic subtypes of CDX2^+^ GC, their different responses to both CPI‐637, and the strategy to overcome 5‐FU resistance in CDX2^+^ REG4^hi^ GC. Data are presented as the means ± SD. Error bars represents for standard deviations. *p < .05, **p < .01, ***p < .0001, *NS*, not significant.

In both CDX2^+^ REG4^hi^ CDX and patient derived xenograft (PDX) in vivo, treatment with CPI‐637 and particularly with the CPI‐637 and 5‐FU combination significantly inhibited tumour growth (Figure 4E and F). Furthermore, CPI‐637 and 5‐FU combination markedly improved mouse survival compared to 5‐FU or CPI‐637 alone (Figure 4E and F). We compared the tumor inhibition rate and found that 5‐FU was more effective in CDX2+ REG4lo GC while CPI‐637 was more effective in CDX2+ REG4hi GC (Figure 2G). The combination did not result in more systemic toxicity or weight loss than 5‐FU alone (Figure 4H and I). Collectively, these data indicate that targeting CBP can overcome 5‐FU resistance and thus provide a therapeutic option for efficacy improvement of 5‐FU‐based chemotherapy in CDX2^+^ REG4^hi^ GC (Figure 4J). This study provides crucial insights into the molecular mechanisms driving chemoresistance and offers potential for clinical translation.

## Supporting information



Supporting information

Supporting information

Supporting information

Supporting information
