# Supplementary figures and images for: Targeting CBP revers chemoresistance to 5‐FU of CDX2/REG4 double‐positive gastric cancer
Source: Clin Transl Med. 2024 Oct 25;14(11):e70069. doi: 10.1002/ctm2.70069 (PMC11511671; doi:10.1002/ctm2.70069)

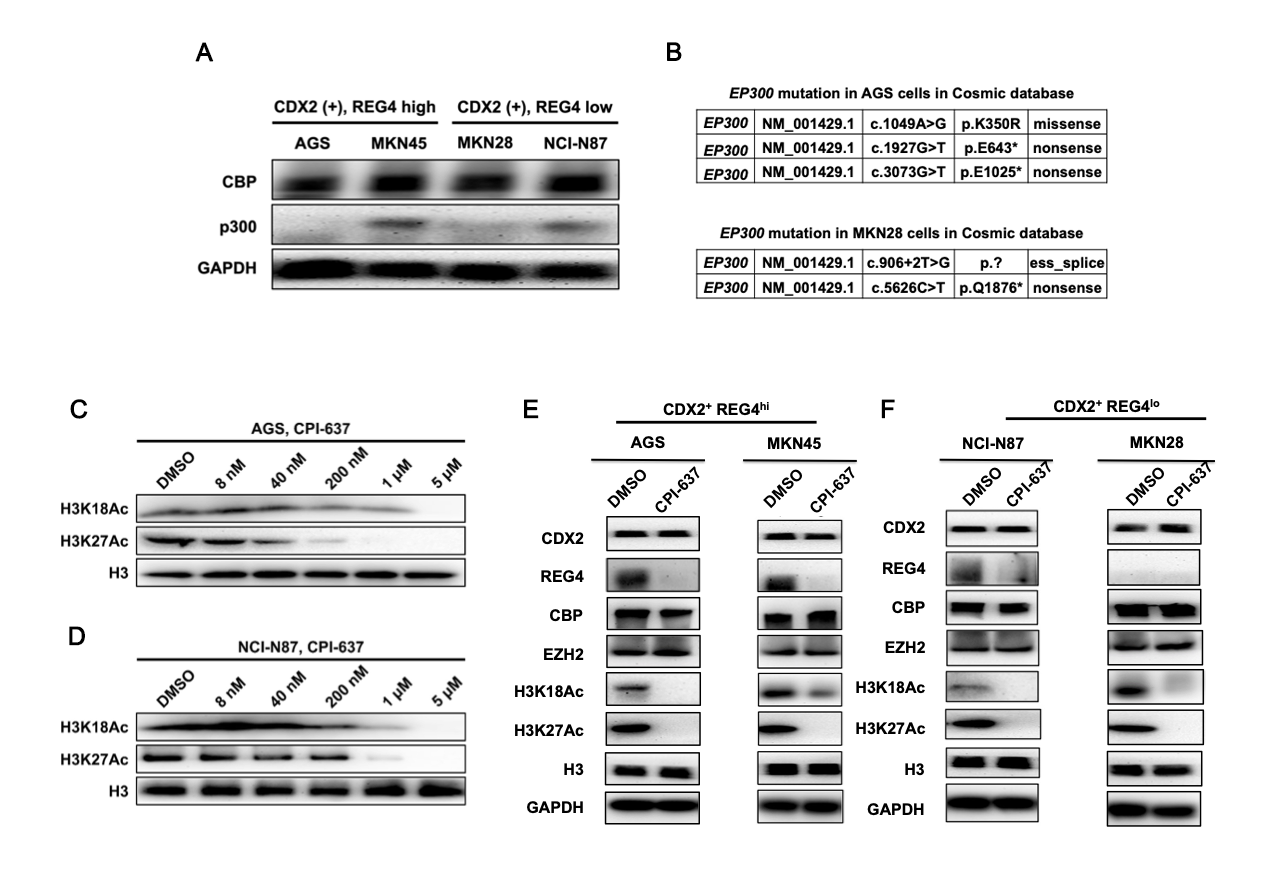

Supplement: Supplementary file 1 — Supporting information [file CTM2-14-e70069-s004.tif]

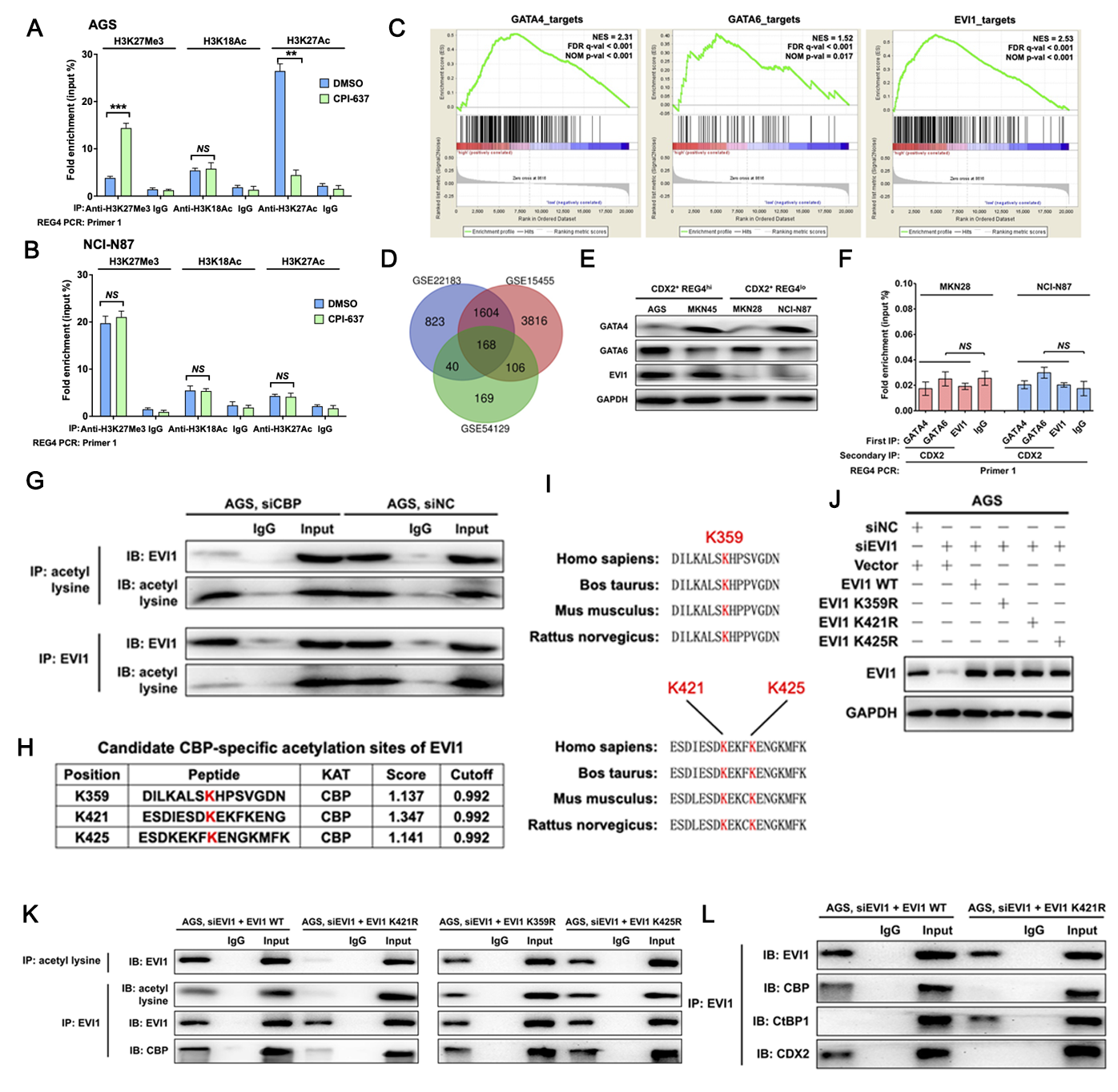

Supplement: Supplementary file 2 — Supporting information [file CTM2-14-e70069-s001.tif]
