# Supplementary material for: Targeting CBP revers chemoresistance to 5‐FU of CDX2/REG4 double‐positive gastric cancer
Source: Clin Transl Med. 2024 Oct 25;14(11):e70069. doi: 10.1002/ctm2.70069 (PMC11511671; doi:10.1002/ctm2.70069)
Supplement: Supplementary file 3 — Supporting information [file CTM2-14-e70069-s002.docx]

**Supplemental materials**

**Methods**

**Patients and Tumors**

Three independent GC cohorts were enrolled in this study. The Ruijin array cohort and the Ruijin IHC cohort were from Shanghai Ruijin Hospital. The remaining cohort was the TCGA cohort. The Ruijin array cohort included 111 GC samples (108 with overall survival (OS) data) that were randomly collected from GC patients undergoing gastrectomy from 2006 to 2008. This cohort was used as the primary training set to identify prognosis-related genes in CDX2 high-expression GC by performing a DEG analysis based on a two-tailed unpaired t-test with standards of fold change (FC) > 2 and *P* < 0.05. The Ruijin IHC cohort (FFPE specimens, n = 161, all with OS information) served as the internal validation set for the IHC staining. GC tissue samples in this cohort were randomly collected from GC patients undergoing gastrectomy from 2001 to 2003. Follow-up was terminated on 31 December 2017 for the Ruijin array cohort, and 31 December 2010 for the Ruijin IHC cohort. For the two Ruijin cohorts, clinicopathological data were collected and pathological tumor staging was determined according to the AJCC TNM classification. None of these patients had received preoperative radiotherapy or chemotherapy. In addition, the TCGA cohort was used as an external validation set. There were 384 GC samples in the TCGA cohort with RNA-sequencing expression profiling data and OS information, of which 205 also had disease-free survival (DFS) information and 47 were known to have received 5-FU-based adjuvant chemotherapy. OS and DFS in this study were defined as previously reported. This study was approved by the ethics committee of Shanghai Ruijin Hospital.

**Immunohistochemistry (IHC) Staining**

For IHC staining, all staining steps were performed according to a standard LSAB protocol (Dako). The primary antibodies were rabbit antibody against human CDX2 (Cell Signaling Technology, CST) and goat antibody against human REG4 (R&D). The secondary antibodies were biotinylated swine anti-rabbit antibody (Dako) or anti-goat antibody (Dako). The protein expression levelof CDX2 was detected in the nuclei, while REG4 was detected in the membrane and extracellular matrix. Positivity for more than 10% of the cancer cells was considered positive expression of the proteins. Omission of the primary antibodies was used as a negative control.

**Cell** **Proliferation Assays**

GC cell proliferation at indicated time under the indicated treatment was measured with the Cell Counting Kit-8 (CCK-8) (Dojindo) according to the manufacturer’s instructions. In the inhibitor screening experiments, GC cells were treated with the indicated epigenetic small molecule inhibitor with a final concentration equal to the IC_50_ as previously established in the literature (detailed information in Supplementary Table S3) or an equal volume of vehicle control (0.1% DMSO) for 72 hours before the measurement of proliferation. In the IC_50_ detection experiments, the IC_50_ values were calculated according to the dose-response curve fitted according to the four-parameter method. For the assessment of the combination effects in vitro, cells were treated with 5-FU, GSK343, CPI-637, or a combination of two of the drugs at gradient concentrations for 72 hours, and then the Chou-Talalay combination index (CI) was calculated using CompuSyn software. CI < 0.9, 0.9~1.1 and > 1.1 were considered synergism, addition and antagonism, respectively.

**siRNAs, Plasmid Vectors and Transfection**

Transient knockdown of CDX2, CBP, and EVI1 in GC cells was performed using siRNAs. A pool of siRNAs targeting human CDX2 was obtained from Santa Cruz Biotechnology (Cat# sc-43680). Human CBP-specific siRNA targeted the 5’-CCCGATAACTTTGTGATGTTT-3’ sequence (NM_004380, 3’UTR region). Human EVI1-specific siRNA targeted the 5’-TGCAGGGTCACTCATCTAAAG-3’ sequence (NM_005241, 3’UTR region). A scrambled siRNA (siNC) (5’-AACTCGCTGAGATTTCGTTAT-3’) served as a negative control to control specificity. CBP and EVI1 siRNAs were synthetized by Shanghai Obio Technology Company. siRNA (100 nM) targeting a gene of interest or siNC was transfected using a Lipofectamine 2000 transfection reagent kit (ThermoFisher Scientific) according to the manufacturer’s instructions.

Full-length wild-type human REG4 and EVI1 were cloned into a pcDNA3.1 plasmid vector for overexpression. Human REG4 3’UTR-specific shRNA targeting the 5’-GCAAGAATCAAGATTCTGCTA-3’ sequence or the scrambled shRNA (shNC) targeting 5’-AACTCGCTGAGATTTCGTTAT-3’ was used for endogenous REG4 knockdown. The three EVI1 mutants (EVI1 K359R, EVI1 K421R and EVI K425R) were generated by creating a mutation in the EVI1 sequence. Specifically, the sequence encoding lysine 359, lysine 421, or 425 in the EVI1 sequence was mutated to a sequence encoding arginine. For stable transfection, GC cells were transfected with 4 μg of plasmid using Lipofectamine 2000. Stable transfects were obtained with 800 μg/ml G418 for 4 weeks.

**Reverse-Transcription Quantitative PCR (RT-qPCR)**

RT-qPCR assays were performed according to the protocol. The primers for RT-qPCR were as follows: *REG4* forward, 5’-TGGAGCAGCAACGAATGC-3’, reverse, 5’-TGCTCTATGGTCGGTACTTGCA-3’; *GAPDH* forward, 5’-TCGGAGTCAACGGATTTGGTC-3’, reverse, 5’-ATGGAATTTGCCATGGGTGGA-3’.

**Western Blot**

Protein extracts quantified using a BCA Protein Assay Kit (Pierce) were resolved through 8%~15% SDS-PAGE, transferred to PVDF membranes, and immunoblotted using rabbit antibodies against human CDX2 (1:1000, CST), CBP (1:1000, CST), histone H3K27Ac (1:1000, CST), histone H3K27Me3 (1:1000, CST), histone H3K18Ac (1:1000, CST), histone H3K9Me2 (1:1000, CST), histone H3 (1:1000, CST), EVI1 (1:1000, CST), GATA4 (1:1000, CST) and GATA6 (1:1000, CST), or mouse antibodies against human REG4 (1:1000, Abcam) and GAPDH (1:10000, ProteinTech). Peroxidase-conjugated anti-mouse or rabbit IgG antibodies (1:5000, ProteinTech) were used as secondary antibodies, and the antigen-antibody reaction was visualized with the Pierce ECL kit (Thermo).

**Co-Immunoprecipitation (****Co-IP)**

For immunoprecipitation or Co-IP experiments, cell lysates were mixed with an equal amount of Co-IP buffer (Millipore). Primary rabbit antibodies against CDX2 (CST), CBP (CST), EVI1 (CST), acetylated-lysine (CST), or an isotype-matched normal rabbit IgG (CST) were added to the lysates, followed by an incubation at 4ºC on a rotary device for 2 hours and blocking with protein A + G beads (Millipore) at 4ºC overnight. Then, the beads were collected by centrifugation and used for western blot analysis.

**Chromatin Immunoprecipitation (ChIP)** **and Sequential ChIP Assay**

ChIP assays were performed utilizing the SimpleChIP^®^ Plus Enzymatic Chromatin IP Kit (Magnetic Beads) (CST) according to the manufacturer’s protocol. The following antibodies were used: rabbit antibodies against CDX2 (CST), CBP (CST), histone H3K27Ac (CST), histone H3K27Me3 (CST), histone H3K18Ac (CST), EVI1 (CST), GATA4 (CST) and GATA6 (CST), or an isotype matched normal rabbit IgG (CST). For sequential ChIP assays, when the DNA-protein complexes were eluted followed by centrifugation, the supernatant was collected and diluted to 500:1 with IP buffer and subjected to the ChIP procedure again. For ChIP and sequential ChIP assays, the purified DNA was then subjected to SYBR Green Quantitative PCR using the following two primer pairs: primer pair 1 for human *REG4* promoter, forward, 5’-GGAGAGGTTCTTTTCCTGGCTAG-3’, reverse, 5’- GCAACCAAGACTCTAAGGGCC-3’ and primer pair 2 for *REG4* enhancer, forward, 5’-GTTTCCACAAAAATATTCGCCCC-3’, reverse, 5’-TCTCTCTCCATAAGTCCACAGA-3’. The enrichment of the indicated protein on the *REG4* transcriptional regulatory regions was shown as a percentage of the input (% input).

**Mouse Xenograft Model and Subcutaneous Tumor Therapy**

GC cells (2 × 10^6^ cells per mouse in 100 μl sterile 1 × PBS) were injected subcutaneously into the flank of 6-week-old female BALB/c-nu/nu mice according to a protocol approved by the Ethical Committee on Animal Experiments at Ruijin Hospital. Seven days after injection for AGS, MKN45 and MKN28 cells, or 14 days after injection for NCI-N87 cells, the mice were randomized to treatment groups, and treatment was immediately initiated as indicated. GSK343, CPI-637 and 5-FU were administered at 25 mg/kg, 25 mg/kg and 20 mg/kg per dose (intraperitoneal) and three doses per week, respectively. An equal volume of vehicle (DMSO) was used as a control. Tumor size and body weight were measured twice a week. The volume of the tumors (V) was calculated as V = L × S^2^ /2 (mm^3^), where L and S represent the largest and the smallest diameters (mm), respectively. For survival analysis, the end-point was when the tumor area reached 100 mm^2^ (calculated as L × S). For experiments with the generation of tumor growth curves, mice were euthanized 28 days after inoculation with AGS, MKN45 and MKN28 cells, or 35 days after inoculation of NCI-N87 cells. In experiments with survival analyses, the mice were euthanized when the tumor area reached the endpoint otherwise until 60 days after inoculation.

For the assessment of the combined effects of 5-FU and CPI-637 in vivo, the Bliss independence model was used as previously reported. Briefly, the growth inhibition rates of 5-FU and CPI-637 treatment alone compared to the vehicle treatment alone were labeled as A and B, respectively. The Bliss expectation was determined as (A + B) – A × B. A significantly higher real growth inhibition rate of the combination therapy than that calculated as the Bliss expectation was considered a robust synergistic effect, while a significantly lower real growth inhibition rate and a rate comparable to the Bliss expectation were determined as a robust antagonistic effect and an additive effect, respectively.

**Gene Set Enrichment Analysis (GSEA)**

Gene set enrichment analysis (GSEA) was performed for the gene expression profiles between CDX2^+^ REG4^hi^ and CDX2^+^ REG4^lo^ GC tissues in GSE54129 using gene sets representing potential targets of regulation by transcription factors (TFs) (the MSigDB, C3 collection: motif gene sets, http://software.broadinstitute.org/gsea/msigdb/genesets.jsp?collection=C3). Standard settings with 1000 permutations were used in this study. Gene sets with a *P* value < 0.05 were considered significantly enriched.

**Statistical Analysis**

The detailed information regarding the statistical analysis is described in the Supplementary Materials and Methods.

**Supplementary Figure 1.** Basal levels of CBP and p300 and mutation information for *EP300* in the two subtypes of CDX2^+^ GC cells. **A**, Western blot of CBP and p300 and the loading control GAPDH in the two subtypes of CDX2^+^ GC cells. **B**, Somatic mutation information for *EP300* in AGS and MKN28 cells from the COSMIC database. **C** and **D**, Western blot of the indicated proteins upon treatment with the indicated doses of CPI-637 or 0.1% DMSO for 72 hours in AGS (**C**) and NCI-N87 (**D**) cells. **E** and **F**, Western blot of the indicated proteins upon treatment with 5 μM CPI-637 or 0.1% DMSO for 72 hours in CDX2^+^ REG4^hi^ AGS and MKN45 cells (**E**) and CDX2^+^ REG4^lo^ NCI-N87 and MKN45 (**F**) cells.

**Supplementary Figure 2.** CBP-catalyzed EVI1 K421 acetylation is required for the selective recruitment of CBP to the *REG4* promoter by CDX2 in CDX2^+^ REG4^hi^ GC cells. **A-B,** Changes in H3K27Me3, H3K27Ac and H3K18Ac after CPI-637 treatment. ChIP experiments were performed in AGS (**A**) and NCI-N87 (**B**) cells. Cells were treated for 24 hours, and then protein-DNA was cross-linked and sequentially immunoprecipitated by two antibodies as indicated. The purified DNA fragments were amplified and quantified by qPCR using the promoter primer pairs (Primer 1). Enrichment of the indicated protein on *REG4* transcriptional regulatory regions was shown as a percentage of the input (% input). IgG, immunoglobulin G. Data are presented as the means ± SD. Data represent three replicates; ***, *P* < 0.001, *NS*, not significant, two-tailed unpaired Student’s *t* test. **C,** Overview of the obtained most significant TF enrichment plots (FDR q < 0.001) by GSEA analysis for the gene expression profiles between the two subtypes of CDX2^+^ GC in GSE54129 (using “C3 collection: motif gene sets” from the MSigDB). **D,** Venn diagram showing the overlap of the significantly (FC > 2 and *P* < 0.05) DEGs between the two subtypes of CDX2^+^ GC in GSE54129 and two independent GC cell line cohorts, GSE15455 and GSE22183. In total, 168 DEGs common among the three datasets were identified and are listed in Supplementary Table S3. **E,** Western blot of GATA4, GATA6, EVI1 and the loading control GAPDH in the two subtypes of CDX2^+^ GC cells. **F**, Enrichment of the CDX2, GATA4, GATA6 and EVI1 in the *REG4* promoter in CDX2^+^ REG4^lo^ GC MKN28 and NCI-N87 cells were detected by sequential ChIP using the two indicated antibodies. Enrichment of the indicated protein in the *REG4* promoter region is shown as a percentage of the input (% input). **G,** Co-immunoprecipitation (Co-IP)-Western blot for detecting the acetylated EVI1 and general cell protein acetylation level in AGS cells transiently transfected with siRNA targeting CBP (siCBP) or a negative control siRNA (siNC) for 72 hours. IgG, immunoglobulin G. IB, immunoblot. **H**, Candidate CBP KAT-specific lysine acetylation sites (K359, K421 and K425) in the region spanning amino acids 283 to 514 of EVI1 using the GPS-PAIL algorithm. **I**, Alignment of EVI1 K359, K421 and K425-centered amino acid sequences among different species. **J**, AGS cells were transiently transfected with a 3’UTR-targeting siRNA against EVI1 (siEVI1) alone, siEVI1 together with a plasmid expressing wild-type EVI1 (EVI1 WT), EVI1 359 Lys-to-Arg mutant (EVI1 K359R), EVI1 K421R mutant, or EVI1 K425R mutant, and the negative control siRNA (siNC) + the empty plasmid (vector) for 72 hours. Then, EVI1 and the loading control GAPDH were detected by Western blot. **K**, Co-IP-Western blot for detecting the acetylated EVI1 and the physical interactions between EVI1 and CBP in AGS cells transiently transfected with siEVI1 together with a plasmid expressing EVI1 WT, EVI1 K359R mutant, EVI1 K421R mutant or EVI1 K425R mutant. **L**, Co-IP-Western blot for detecting the physical interactions between EVI1 and CDX2/CBP/CtBP1 in AGS cells transiently transfected with siEVI1 together with a plasmid expressing EVI1 WT or EVI1 K421R mutant. Data are presented as the means ± SD. Data represent three replicates; **, *P* < 0.01, ***, *P* < 0.001, *NS*, not significant, two-tailed unpaired Student’s *t* test.

**Ethics approval and consent to participate**

All procedures of human and mouse experiments were approved by Ethics Committee of Shanghai Ruijin Hospital, Shanghai Jiao Tong University School of Medicine, Shanghai, China (Approval No.2017-6)

**Consent for publication**

Not applicable

**Data Availability Statement**

Not applicable

**Competing interests**

The authors declare that they have no competing interests.

**Funding**

This study was supported by grants from the National Natural Science Foundation of China (No. 81871902, No. 81871904, and No. 82072605) Shanghai Sailing Program (No.22YF1426100) and Shanghai Anticancer Association EYAS PROJECT (No.SACA-CY21B06).

**Authors’ Contributions**

Conception and design: B. Liu, C. Yan, F. Yuan, and Z. Fan

Development of methodology: Z. Fan, F. Li, X. Jiang

Acquisition of data (provided animals, acquired and managed patients, provided facilities, etc.): Z. Fan, F. Li, X. Jiang, J. Li, B. Y, T. Pan, M. Zang,J. Hou, W. Liu, C. Yan, W. Liu, M. Yan, F. Yuan

Analysis and interpretation of data (e.g., statistical analysis, biostatistics, computational analysis):

Z. Fan, F. Li X. Jiang

Writing, review, and/or revision of the manuscript: Z. Fan, F. Yuan, B. Liu

Administrative, technical, or material support (i.e., reporting or organizing data, constructing databases): Z. Fan, F. Li, X. Jiang, J. Li, B. Yu, T. Pan, M. Zang, C. Yan, W. Liu, C. Li, M. Yan, L. Su, Z. Zhu, F. Yuan

Study supervision: B. Liu

**Acknowledgements**

Not applicable
