# Supplementary material for: Targeting CBP revers chemoresistance to 5‐FU of CDX2/REG4 double‐positive gastric cancer
Source: Clin Transl Med. 2024 Oct 25;14(11):e70069. doi: 10.1002/ctm2.70069 (PMC11511671; doi:10.1002/ctm2.70069)
Supplement: Supplementary file 4 — Supporting information [file CTM2-14-e70069-s003.docx]

**Supplementary Tables and Supplementary Table Legends**

**Supplementary Table S1, related to Figure 1. Associations between the two molecular subtypes of CDX2-positive GC and patient characteristics in the Ruijin IHC cohort (n = 113).**

| **Characteristics** | **CDX2 (+) & REG4 (+)** | **CDX2 (+) & REG4 (-)** | ***P* value** |
| --- | --- | --- | --- |
| *N* | 60 (53.1%) | 53 (46.9%) |  |
| **Age** | 55.6 ± 7.7 | 58.3 ± 11.5 | 0.1413 |
| **Gender** |  |  |  |
| Male | 36 (60.0%) | 38 (71.7%) | 0.1918 |
| Female | 24 (40.0%) | 15 (28.3%) |  |
| **Tumor size** |  |  |  |
| ≤ 5 cm | 28 (46.7%) | 37 (69.8%) | 0.0130 |
| > 5cm | 32 (53.3%) | 16 (30.2%) |  |
| **Histological grade** |  |  | 0.0080 |
| Grade 1~2 | 16 (26.7%) | 27 (50.9%) |  |
| Grade 3 | 44 (73.3%) | 26 (49.1%) |  |
| **pT (AJCC 7^th^ ed.)** |  |  | 0.2090 |
| T1~2 | 49 (81.7%) | 38 (71.7%) |  |
| T3~4 | 11 (18.3%) | 15 (28.3%) |  |
| **pN (AJCC 7^th^ ed.)** |  |  | 0.3025 |
| N0 | 27 (45.0%) | 29 (54.7%) |  |
| N1~3 | 33 (55.0%) | 24 (45.3%) |  |
| **pTNM (AJCC 7^th^ ed.)** |  |  | 0.2310 |
| I | 25 (%) | 25 (%) |  |
| II | 10 (%) | 13 (%) |  |
| III | 22 (%) | 15 (%) |  |
| IV | 3 (%) | 0 (%) |  |

Abbreviations: pT, pathological staging of the primary tumor; pN, pathological staging of the regional lymph nodes; pTNM, overall pathological staging.

For age, unpaired t test was used. For pTNM stage, Linear trend test was used. For all other variables, Chi-square test or Fisher's exact test was used.

**Supplementary Table S2.** **Univariate and multivariate analyses of the association of the clinicopathological characteristics with the two molecular subtypes with regard to the overall survival of patients with CDX2-positive GC in the Ruijin IHC cohort (n = 113).**

| **Characteristics** | **Univariate analyses** | |  | **Multivariate analyses** | |
| --- | --- | --- | --- | --- | --- |
|  | **HR (95% CI)** | ***P* value** |  | **HR (95% CI)** | ***P* value** |
| Age (> 60 vs ≤ 60) | 1.03 (0.53 to 1.99) | 0.94 |  |  |  |
| Gender (female vs male) | 1.51 (0.78 to 3.12) | 0.21 |  |  |  |
| Tumor size (> 5cm vs ≤ 5cm) | 1.56 (1.03 to 3.26) | **0.015** |  | 1.13 (0.95 to 1.34) | 0.17 |
| Histological grade (grade 1~2 vs grade 3) | 0.97 (0.49 to 1.90) | 0.93 |  |  |  |
| Borrmann classification (III + IV vs I + II) | 1.12 (0.42 to 3.01) | 0.81 |  |  |  |
| Location of tumor |  |  |  |  |  |
| Cardia vs antrum | 1.66 (0.39 to 7.09) | 0.49 |  | 1.31 (0.55 to 3.17) | 0.54 |
| Body vs antrum | 2.48 (1.29 to 6.00) | **0.0089** |  | 2.83 (0.19 to 42.03) | 0.45 |
| Whole, multicentric vs antrum | 10.38 (139.3 to 43993) | **< 0.0001** |  | 0.93 (0.27 to 3.18) | 0.90 |
| AJCC TNM stage (7^th^ ed.) (III + IV vs I + II) | 3.83 (2.31 to 9.14) | **< 0.0001** |  | 3.76 (1.76 to 8.02) | **0.0006** |
| CDX2 (+) & REG4 (+) vs CDX2 (+) & REG4 (-) | 8.22 (2.99 to 10.76) | **< 0.0001** |  | 2.67 (1.55 to 3.89) | **0.013** |

Both univariate and multivariate analyses used the Cox proportional hazards regression model. The clinicopathological variables in multivariate analyses were adopted for their prognostic significance by univariate analyses.

**Supplementary Table S3. Epigenetic small molecule inhibitor panel used in this study.**

| **Inhibitor name** | **Target** | **Type of epigenetic regulator** | **Dose [nM]** | **PMID** |
| --- | --- | --- | --- | --- |
| SGI-1027 | DNMT1, 3A, 3B | Epigenetic “writer”,  DNA methyltransferase | 8000 | 19417133 [^1^](#_ENREF_1) |
| BIX 01294 | G9a | Epigenetic “writer”,  histone methyltransferase | 2700 | 17289593 [^2^](#_ENREF_2) |
| MM-102 | MLL1 | Epigenetic “writer”,  histone methyltransferase | 400 | 23210835 [^3^](#_ENREF_3) |
| GSK343 | EZH2 | Epigenetic “writer”,  histone methyltransferase | 40 | 24900432 [^4^](#_ENREF_4) |
| SGC707 | PRMT3 | Epigenetic “writer”,  arginine methyltransferase | 31 | 25728001 [^5^](#_ENREF_5) |
| MS023 | PRMT1, 3, 4, 6, 8 | Epigenetic “writer”,  arginine methyltransferase | 119 | 26598975 [^6^](#_ENREF_6) |
| UNC0379 | SETD8 | Epigenetic “writer”,  N-lysine methyltransferase | 7900 | 25032507 [^7^](#_ENREF_7) |
| MG149 | TIP60, MOF | Epigenetic “writer”,  histone acetyltransferase | 74000 | 22100137 [^8^](#_ENREF_8) |
| CPI-637 | CBP/EP300 | Epigenetic “writer”,  lysine acetyltransferase | 51 | 27190605 [^9^](#_ENREF_9) |
| JIB-04 | JARID1A, JMJD2A, 2B, 2C, 2D, 2E, 3 | Epigenetic “eraser”,  Jumonji histone demethylase | 1100 | 23792809 [^10^](#_ENREF_10) |
| ML324 | JMJD2 | Epigenetic “eraser”,  Jumonji histone demethylase | 920 | 24260783 [^11^](#_ENREF_11) |
| Panobinostat | HDAC | Epigenetic “eraser”,  histone deacetylase | 5 | 18349321 [^12^](#_ENREF_12) |
| GSK J4 HCl | JMJD3, UTX | Epigenetic “eraser”,  histone demethylase | 60 | 22842901 [^13^](#_ENREF_13) |
| PFI-1 | BRD2, 4 | Epigenetic “reader” | 220 | 23576556 [^14^](#_ENREF_14) |
| BI-7273 | BRD9 | Epigenetic “reader” | 19 | 26914985 [^15^](#_ENREF_15) |
| PFI-3 | SMARCA2, 4 | Epigenetic “reader” | 110 | 26139243 [^16^](#_ENREF_16) |
| I-BET-762 | BET | Epigenetic “reader” | 35 | 21068722 [^17^](#_ENREF_17) |

To lessen potential off-target effects, dose of each small molecule inhibitor used in this study was the IC_50_ of their intended target as determined according to previously published literature.

**Supplementary Table S4. List of the 168 DEGs common among GSE54129, GSE15455 and GSE22183.**

| **Gene Symbol** | **Description** |
| --- | --- |
| REG4 | regenerating family member 4 |
| C9orf152 | chromosome 9 open reading frame 152 |
| GALNT3 | polypeptide N-acetylgalactosaminyltransferase 3 |
| STYK1 | serine/threonine/tyrosine kinase 1 |
| XK | X-linked Kx blood group |
| TM4SF4 | transmembrane 4 L six family member 4 |
| F2RL1 | F2R like trypsin receptor 1 |
| B3GNT7 | UDP-GlcNAc:betaGal beta-1,3-N-acetylglucosaminyltransferase 7 |
| KIAA1324 | KIAA1324 |
| MLF1 | myeloid leukemia factor 1 |
| POF1B | POF1B, actin binding protein |
| SAA2 | serum amyloid A2 |
| TMEM238L | ncRNA, LINC00675 |
| CYP3A5 | cytochrome P450 family 3 subfamily A member 5 |
| MUC2 | mucin 2, oligomeric mucus/gel-forming |
| UPK1B | uroplakin 1B |
| SDR16C5 | short chain dehydrogenase/reductase family 16C member 5 |
| APOA2 | apolipoprotein A2 |
| LGALS4 | galectin 4 |
| CBS | cystathionine-beta-synthase |
| IL20RA | interleukin 20 receptor subunit alpha |
| NPNT | nephronectin |
| ZBTB7C | zinc finger and BTB domain containing 7C |
| LAMA3 | laminin subunit alpha 3 |
| AP1S3 | adaptor related protein complex 1 subunit sigma 3 |
| CKMT1A | creatine kinase, mitochondrial 1A |
| CEACAM5 | carcinoembryonic antigen related cell adhesion molecule 5 |
| MPP6 | membrane palmitoylated protein 6 |
| TACSTD2 | tumor associated calcium signal transducer 2 |
| PPP1R36 | protein phosphatase 1 regulatory subunit 36 |
| SPINK4 | serine peptidase inhibitor, Kazal type 4 |
| ZBTB10 | zinc finger and BTB domain containing 10 |
| ADIRF | adipogenesis regulatory factor |
| CRIP1 | cysteine rich protein 1 |
| NMUR2 | neuromedin U receptor 2 |
| SOSTDC1 | sclerostin domain containing 1 |
| H19 | H19, imprinted maternally expressed transcript |
| SLC27A2 | solute carrier family 27 member 2 |
| ALB | albumin |
| SLC4A4 | solute carrier family 4 member 4 |
| APOC1 | apolipoprotein C1 |
| HYAL1 | hyaluronidase 1 |
| AGR2 | anterior gradient 2, protein disulphide isomerase family member |
| ST6GALNAC1 | ST6 N-acetylgalactosaminide alpha-2,6-sialyltransferase 1 |
| PSPH | phosphoserine phosphatase |
| DEPDC7 | DEP domain containing 7 |
| AKR1B10 | aldo-keto reductase family 1 member B10 |
| CD55 | CD55 molecule (Cromer blood group) |
| POU2AF1 | POU class 2 associating factor 1 |
| TCIM | transcriptional and immune response regulator |
| FOLR1 | folate receptor 1 |
| CST1 | cystatin SN |
| DSC2 | desmocollin 2 |
| RASEF | RAS and EF-hand domain containing |
| LRRC31 | leucine rich repeat containing 31 |
| CLDN7 | claudin 7 |
| CDH17 | cadherin 17 |
| KCNK1 | potassium two pore domain channel subfamily K member 1 |
| TFF2 | trefoil factor 2 |
| MAP7D2 | MAP7 domain containing 2 |
| SLC6A20 | solute carrier family 6 member 20 |
| MUC4 | mucin 4, cell surface associated |
| GSDME | gasdermin E |
| SGPP2 | sphingosine-1-phosphate phosphatase 2 |
| LINC02381 | long intergenic non-protein coding RNA 2381 |
| ENPP5 | ectonucleotide pyrophosphatase/phosphodiesterase 5 (putative) |
| APOBEC1 | apolipoprotein B mRNA editing enzyme catalytic subunit 1 |
| IL1R2 | interleukin 1 receptor type 2 |
| CYP2C18 | cytochrome P450 family 2 subfamily C member 18 |
| SLC1A1 | solute carrier family 1 member 1 |
| NR5A2 | nuclear receptor subfamily 5 group A member 2 |
| MAGEA6 | MAGE family member A6 |
| KLK10 | kallikrein related peptidase 10 |
| GMDS | GDP-mannose 4,6-dehydratase |
| CDKN2A | cyclin dependent kinase inhibitor 2A |
| C1orf116 | chromosome 1 open reading frame 116 |
| TFF3 | trefoil factor 3 |
| GATA4 | GATA binding protein 4 |
| UGT1A3 | UDP glucuronosyltransferase family 1 member A3 |
| KRT23 | keratin 23 |
| GSKIP | GSK3B interacting protein |
| HSD17B2 | hydroxysteroid 17-beta dehydrogenase 2 |
| HMGCS2 | 3-hydroxy-3-methylglutaryl-CoA synthase 2 |
| IGF2BP3 | insulin like growth factor 2 mRNA binding protein 3 |
| CEACAM6 | carcinoembryonic antigen related cell adhesion molecule 6 |
| MAGEA3 | MAGE family member A3 |
| MSMB | microseminoprotein beta |
| ADGRF1 | adhesion G protein-coupled receptor F1 |
| PCDH7 | protocadherin 7 |
| QPRT | quinolinate phosphoribosyltransferase |
| CDS1 | CDP-diacylglycerol synthase 1 |
| SULT1C2 | sulfotransferase family 1C member 2 |
| FCGBP | Fc fragment of IgG binding protein |
| VSIG1 | V-set and immunoglobulin domain containing 1 |
| BEX2 | brain expressed X-linked 2 |
| ANKRD22 | ankyrin repeat domain 22 |
| ADH1C | alcohol dehydrogenase 1C (class I), gamma polypeptide |
| TSPYL5 | TSPY like 5 |
| RPS4Y1 | ribosomal protein S4 Y-linked 1 |
| ZC3H12C | zinc finger CCCH-type containing 12C |
| MEP1A | meprin A subunit alpha |
| PTGER4 | prostaglandin E receptor 4 |
| CAPN8 | calpain 8 |
| DAZ4 | deleted in azoospermia 4 |
| INSM1 | INSM transcriptional repressor 1 |
| CYP2S1 | cytochrome P450 family 2 subfamily S member 1 |
| XIST | X inactive specific transcript |
| CTSE | cathepsin E |
| ITGB6 | integrin subunit beta 6 |
| MACC1 | MET transcriptional regulator MACC1 |
| REG1A | regenerating family member 1 alpha |
| TFAP2A | transcription factor AP-2 alpha |
| RFX6 | regulatory factor X6 |
| KRT19 | keratin 19 |
| SLC39A8 | solute carrier family 39 member 8 |
| F5 | coagulation factor V |
| LYZ | lysozyme |
| NMU | neuromedin U |
| IL1B | interleukin 1 beta |
| PIP5K1B | phosphatidylinositol-4-phosphate 5-kinase type 1 beta |
| SIAE | sialic acid acetylesterase |
| CEACAM1 | carcinoembryonic antigen related cell adhesion molecule 1 |
| TFF1 | trefoil factor 1 |
| BHLHE41 | basic helix-loop-helix family member e41 |
| BCL2L14 | BCL2 like 14 |
| IQGAP2 | IQ motif containing GTPase activating protein 2 |
| C3orf14 | chromosome 3 open reading frame 14 |
| AGR3 | anterior gradient 3, protein disulphide isomerase family member |
| AFAP1-AS1 | AFAP1 antisense RNA 1 |
| CT45A8 | cancer/testis antigen family 45 member A8 |
| TGM2 | transglutaminase 2 |
| FGG | fibrinogen gamma chain |
| MYO5B | myosin VB |
| DSG2 | desmoglein 2 |
| HSPA4L | heat shock protein family A (Hsp70) member 4 like |
| VILL | villin like |
| ACKR4 | atypical chemokine receptor 4 |
| S100A14 | S100 calcium binding protein A14 |
| CCL20 | C-C motif chemokine ligand 20 |
| PCK1 | phosphoenolpyruvate carboxykinase 1 |
| GALNT7 | polypeptide N-acetylgalactosaminyltransferase 7 |
| SSX4B | SSX family member 4B |
| ZG16B | zymogen granule protein 16B |
| ADGRG6 | adhesion G protein-coupled receptor G6 |
| TPD52 | tumor protein D52 |
| MGLL | monoglyceride lipase |
| CD24 | CD24 molecule |
| PLEK2 | pleckstrin 2 |
| SLC28A2 | solute carrier family 28 member 2 |
| LOC101930067 | ncRNA, LOC101930067 |
| FABP1 | fatty acid binding protein 1 |
| CT83 | cancer/testis antigen 83 |
| VNN1 | vanin 1 |
| CDH1 | cadherin 1 |
| MAGEA2 | MAGE family member A2 |
| CA2 | carbonic anhydrase 2 |
| PROM1 | prominin 1, CD133 |
| ACER2 | alkaline ceramidase 2 |
| TSPAN1 | tetraspanin 1 |
| CMBL | carboxymethylenebutenolidase homolog |
| PTK6 | protein tyrosine kinase 6 |
| ETNK1 | ethanolamine kinase 1 |
| MLPH | melanophilin |
| ONECUT2 | one cut homeobox 2 |
| RASSF6 | Ras association domain family member 6 |
| FXYD3 | FXYD domain containing ion transport regulator 3 |
| PCDHA1 | protocadherin alpha 1 |
| MECOM | MDS1 and EVI1 complex locus |
